# Clinical manifestations and associated disorders in children with celiac disease in southern Iran

**DOI:** 10.1186/s12887-020-02162-1

**Published:** 2020-05-27

**Authors:** Mahshid Dehbozorgi, Naser Honar, Maryam Ekramzadeh, Forough Saki

**Affiliations:** 1 Shiraz University of Medical Sciences, Shiraz, Iran; 2 Neonatal Research Center, Shiraz University of Medical Sciences, Shiraz, Iran; 3 Shiraz Endocrinology and Metabolism Research Center, Shiraz University of Medical Sciences, P.O. Box: 71345-1744, Shiraz, Iran

**Keywords:** Celiac disease, Prevalence of clinical manifestation, Southern Iran, T1DM Type 1 Diabetes Melitus

## Abstract

**Background:**

Celiac disease (CD) is an immune mediated inflammatory enteropathy, triggered by gluten exposure in HLA-DQ2 and/or –DQ8 genetics. The presentation of celiac disease in children is changing, with increase in non-classical symptoms. We aim to evaluate the clinical presentations of celiac disease amongst children, diagnosed with CD.

**Methods:**

In this cross sectional study, we investigated the clinical features of 130 celiac patients at hospitals affiliated with Shiraz University of Medical Sciences. We used their hospital charts and conducted an interview with patients and their parents to find out demographic data, symptoms, laboratory, and histopathology findings for Marsh grading.

**Results:**

Celiac disease was detected more amongst females (63.8%). We found that 5.4% of the patients had BMI more than 95th percentile. The most common GI symptoms were abdominal pain, flatulence and constipation. Also, the most common extra intestinal manifestation included bone pain, long term fatigue and anemia. Flatulence, chronic diarrhea, and paresthesia were observed more amongst male participants. The most common comorbidities were type 1 diabetes mellitus and hypothyroidism.

**Conclusion:**

The most common gastrointestinal symptoms amongst our patients were abdominal pain, flatulence and constipation. Furthermore, the most common extra intestinal manifestations included bone pain, long term fatigue and anemia. The most associated comorbidities with CD in our children were type 1 diabetes mellitus and hypothyroidism.

## What is known

- Celiac disease (CD) is an immune-mediated disorder, developed by ingesting gluten in HLA-DQ2 and/or –DQ8 genetics.
- Clinical manifestations of celiac disease vary

## What is new

- The most common GI symptoms in our children with CD, were abdominal pain, flatulence and constipation, and the most common extra intestinal manifestation were bone pain, long term fatigue and anemia.
- The most common associated comorbidities with CD were type 1 diabetes mellitus and hypothyroidism.

## Background

Celiac disease (CD) is an immune-mediated disorder, triggered by ingestion of gluten in people who are genetically susceptible [1]. Celiac is a common disease, its prevalence in European countries (Finland, Italy, Germany and UK) was reported to be approximately 1% [2]. A study in Iran showed that the prevalence of celiac disease amongst Iranian school children was 0.6% [3]. However, most patients remain undiagnosed due to iceberg pattern of celiac clinical symptoms and its various clinical presentations [4]. This means that many patients have silent celiac disease [5]. Its clinical manifestations are gastrointestinal (GI) and non-gastrointestinal symptoms. GI symptoms are presented in typical celiac disease, which are chronic diarrhea, failure to thrive, vomiting, constipation, abdominal distension, and anorexia. Non-gastrointestinal symptoms are seen more in atypical CD patients and older patients. The symptoms include anemia, osteoporosis, muscle wasting, headache, and epilepsy. Also, CD is associated with some autoimmune diseases, such as Type 1 diabetes, autoimmune thyroiditis, and autoimmune hepatitis [6]. A study on the subject of celiac disease amongst a pediatric population showed that extra-intestinal symptoms are even more common than the classical manifestations [1]. Celiac disease is a relatively common cause of chronic diarrhea in Iran, a Middle Eastern country, and is diagnosed in 2–8% of patients with type1 diabetes in Iran, Israel and Saudi Arabia. Many of these countries have a per capita wheat consumption, ranking amongst the highest in the world [7]. Plant foods are the major constituent of the Iranian diet, and Iranians rank as one of the top wheat-consuming people in the world with per capita consumption of up to 160 kg/year. The most significant characteristic of the Iranian diet is the dependency on bread and rice as major energy sources. The percentage of carbohydrate and fat to total energy contribution in the Iranian diet was estimated at 66 and 22%, respectively [8]. Therefore, the gluten-free diet (GFD) presents a real challenge to both patients and clinicians in this region. This is particularly difficult in the absence of any supply of gluten free diet in Middle Eastern countries.

Due to the wide spectrum of clinical manifestation of CD and its association with other autoimmune disorders in one hand, and lack of sufficient data regarding the clinical spectrum of CD in southern Iran; hence, we initiated this study. Understanding the various clinical presentations of CD in our region helps us to make proper diagnosis of those patients with atypical symptoms during early stages, which can help to prevent the risk of long term complications.

## Methods

A cross sectional study was conducted on 130 children under the age of 18 with celiac disease in southern Iran, from May 2018 till August 2019. CD was diagnosed according to the American College of Gastroenterology (ACG) clinical guideline [9], and was confirmed by positive serological test e.g. anti-tissue transglutaminase (TTG) and also by performing upper endoscopy and having positive pathological results. These patients were under treatment in pediatric gastroenterologists in celiac clinics affiliated with Shiraz University of Medical Sciences (SUMS). We used their hospital charts and conducted an interview patients and their parents to find out demographic data, symptoms, laboratory, and histopathology findings for Marsh grading. This study was approved by the Vice-chancellor of researches and the local Ethics Committee of SUMS. After explaining the study objectives, written informed consent was obtained from children’s parents or their guardians.

Inclusion criteria were being diagnosed with celiac disease and age under 18 years. Patients with other chronic diseases, such as chronic renal failure, other gastrointestinal problems and congenital disease were excluded. A subjective data gathering questionnaire was filed by the patients or their parents to evaluate the clinical symptoms. Also, clinical signs, biochemical studies, and results from the upper GI endoscopy were collected from the patients’ records.

Tissue transglutaminase: If TTG IgA titer was> 18 u/ml, it was considered to be positive. The IgA level were checked in all children. If a child had IgA deficiency, but normal anti TTG IgA level, anti TTG IgG was checked [10].

Upper GI endoscopy: To confirm celiac disease, upper gastrointestinal endoscopy and tissue biopsy from duodenum was performed. This is the gold standard to diagnose CD. All biopsy specimens were graded according to the modified Marsh classification [11].

### Statistics

Collected data were analyzed with the Statistical Package for Social Sciences (SPSS), version 21. Normality of data were checked by Kolmogrov-Smirnov test. In normal distributed data, we used Chi-square and independent t-test to compare the qualitative and quantitative data, respectively. In others, Man-Whitney test was used to compare quantitative data. Data are presented as mean ± SD. *P* value < 0.05 was considered to be statistically significant.

## Results

Of the130 children who were enrolled in this study 83(63.8%) were female. The mean age of patients was 9.9 ± 3.2 years (range: 2.5–18 years), Table 1. Weight, height and BMI percentile of the patients were 23.02 ± 25, 27.6 ± 25, and 26.9 ±26.6, respectively. With respect to the aforementioned percentiles, there were no significant differences between males and females, Table 1. Ten patients (7.7%) had family history of CD. The most common GI symptom was abdominal pain (66.2%). Table 2 shows other common GI manifestations as follows: flatulence, chronic constipation, malodor stool, diarrhea, lactose intolerance, gastro esophageal reflux, and vomiting. Also, Table 2 shows the most frequent presentations among the extra intestinal manifestations were as follows: bone pain (53.8%), long term fatigue (49.2%), anemia (41.5%), anxiety disorder, itchy skin rash, headache, hair loss, repeated oral ulcer, paresthesia, and seizure. From all the manifestations, flatulence (*p* value = 0.011), chronic diarrhea (*P* = 0.003), paresthesia in hands and feet (*P* = 0.004) were more prevalent amongst male patients, Table 2.

**Table 1:** Demographic and anthropometric data of patients with celiac disease and its comparison in both genders

Data	Total	Male	Female	***P***-value
Age	9.9 ± 3.2	10.1 ± 3.04	9.8 ± 3.3	0.525
Weight	29.5 ± 13.5	28.6 ± 10.6	30.05 ± 14.9	0.577
Height	132.7 ± 18.2	132.9 ± 16.9	132.5 ± 19	0.905
BMI	16 ± 3.48	15.7 ± 2.2	16.1 ± 4.01	0.518
Anti TTG Ab	148.1 ± 166.2	131.8 ± 185.9	155.2 ± 155.3	0.444
Weight percentile	23.02 ± 25.05	21.1 ± 25.9	24.1 ± 24.5	0.514
Height percentile	27.6 ± 25.1	25.8 ± 28.09	28.6 ± 23.3	0.541
BMI percentile	26.9 ± 26.6	23.9 ± 24.2	28.7 ± 27.9	0.326

**Table 2:** Clinical data of patients with celiac disease and its comparison in both genders

Clinical manifestation	Total	Male	Female	***p***-value
Anemia	54(41.5%)	19(40.4%)	35(42.2%)	0.846
Long term fatigue	64(49.2%)	23(48.9%)	41(49.4%)	0.41
headache	25(19.2%)	9(19.1%)	16(19.3%)	0.986
seizure	2(1.5%)	0	2(2.4%)	0.284
Chronic abdominal pain	86(66.2%)	32(68.1%)	54(65.1%)	0.726
Chronic constipation	54(41.5%)	17(36.2%)	37(44.6%)	0.35
flatulence	61(46.9%)	29(61.7%)	32(38.6%)	0.011
Chronic diarrhea	24(18.5%)	15(31.9%)	9(10.8%)	0.003
Gastrointestinal reflux	15(11.5%)	7(14.9%)	8(9.6%)	0.368
Malodor stool	42(32.3%)	17(36.2%)	25(30.1%)	0.479
vomiting	10(7.7%)	4(8.5%)	6(7.2%)	0.792
Lactose intolerance	18(13.8%)	9(19.1%)	9(10.8%)	0.188
Bone pain	70(53.8%)	24(51.1%)	46(55.4%)	0.632
paresthesia	14(10.8%)	10(21.3%)	4(4.8%)	0.004
Itchy skin rash	29(22.3%)	7(14.9%)	22(26.5%)	0.127
Hair loss	20(15.4%)	4(8.5%)	16(19.3%)	0.102
Recurrent oral ulcer	17(13.1%)	7(14.9%)	10(12%)	0.644
Anxiety disorders	43(33.1%)	17(36.2%)	26(31.3%)	0.573

The most common associated comorbidities with CD were diabetes mellitus type 1(15.4%), hypothyroidism (7.7%), down syndrome (1.5%), attention deficit hyperactivity disorder (ADHD) (1.5%) and nephrolithiasis (0.8%). In total, 3.1% of the patients had elevated liver enzyme, which was more common amongst male patients (*P* = 0.007), Table 3.

**Table 3:** Associated disorders with celiac disease in studied patients

Associated disease	Total	Male	Female	***P***-value
Type 1 Diabetes mellitus	20(15.4%)	8(17%)	12(14.5%)	0.697
Hypothyroidism	10(7.7%)	5(10.6%)	5(6%)	0.343
Down syndrome	2(1.5%)	2(4.3%)	0	0.058
Elevated liver enzyme	4(3.1%)	4(8.5%)	0	0.007
Nephrolithiasis	1(0.8%)	1(2.1%)	0	0.182
ADHD	2(1.5%)	2(4.3%)	0	0.056

Body Characteristics: upon evaluating celiac disease on children growth rate, 30.8% had weight percentile ≤ 3 and 4.6% had more than 95th percentile. Also, 21.5% had height percentile ≤ 5 and 3.8% had more than 95th percentile. Out of the total, 29.2% had BMI less than 5th percentile and 5.4% had BMI more than 95th percentile. Weight percentile and BMI was not different amongst both genders, but males were significantly shorter than females (P = 0.038). Table 4 shows the details.

**Table 4:** Body characteristics of children with celiac disease and their comparison in both gender

Variables	Weight percentile			Height percentile			BMI percentile		
	Female	Male	Total	Female	male	Total	Female	Male	Total
**≤5%**	25.3%	40.4%	30.8%	15.7%	31.9%	21.5%	28.9%	29.8%	29.2%
**5–95%**	71.1%	53.2%	64.6%	81.9%	61.7%	74.6%	65.1%	66%	65.4%
**≥ 95%**	3.6%	6.4%	4.6%	2.4%	6.4%	3.8%	6%	4.3%	5.4%
**P value**	0.121			0.038 ***∗***			0.911		

* Significant P value showed the comparison of related percentiles in both sex

In this study, histopathology evaluation of patients showed that 4.8% have Marsh 2, 33.3% Marsh 3a, 32.4% Marsh 3b and 29.5% have Marsh 3c. There was no association between gender and Marsh staging (*p* value: 0.241). There was no association between Marsh staging and weight, height, or BMI percentile.

After evaluating the association between clinical manifestations of CD and Marsh staging, marsh was only associated with long term fatigue (p value: 0.011). In addition to Anti TTG Ab titer had no association with clinical manifestations, and was merely associated with weight percentile in female gender (p = 0.043). Although the mean Anti TTG Ab titer in Marsh 2 was 113.5 ± 68.5 and in Marsh 3 was 155.8 ± 180, the difference was not statistically significant (p = 0.604). In patients with Marsh 2, the mean weight percentile was 11.8 ± 4, and in Marsh 3a, 3b, 3c, weight percentile was 24 ± 25.4, but statistically there was no association between Marsh staging and weight percentile (p = 0.288).

In patients with Marsh 2, BMI percentile was 14.4 ± 14.7, and in Marsh 3a, 3b, 3c it was 28 ± 26.5, but similarly, there was no association between Marsh and BMI percentile (p = 0.259), which was probably due to few number of children with Marsh 2 stage.

**Fig. 1:**
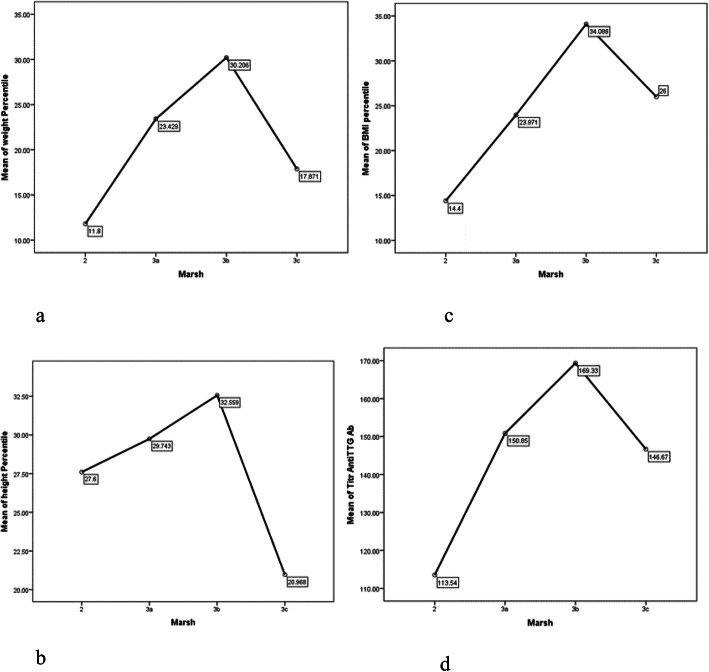
Anthropometric indices include weight(**a**), height(**b**), and BMI percentile(**c**) and anti TTG antibody(**d**) in different Marsh stages of children with celiac disease

Height percentile in patients with Marsh 3c was 20.96 ± 16.8, and in Marsh 2, 3a, 3b was 30.9 ± 26.6; hence, height in Marsh 3c was shorter in comparison with other children (p = 0.009). Figure 1 shows the details.

## Discussion

This retrospective study provides data on clinical manifestation of Celiac disease amongst children in southern Iran. It showed that the most common GI symptoms were abdominal pain, flatulence and constipation, and the most common extra intestinal manifestation were bone pain, long term fatigue and anemia. The most associated comorbidities with CD are type 1 diabetes mellitus and hypothyroidism. From all the manifestations, flatulence, chronic diarrhea, paresthesia in hands and feet were observed more amongst male patients. One study evaluating the gender effect on manifestation of CD in New York showed that men had lower bone density at radius and lower total cholesterol and had indirect evidence for more malabsorptive disorders [12]. We could not find any related article in Iran, showing any association between clinical manifestation of celiac disease and gender. In one study on GI and non-GI presentation in patients with CD in Italy, Romania and Iran found no correlation between clinical manifestation and gender, but in the mentioned countries there were more female patients [13].

In the present study, it was found that celiac disease was detected more amongst females (36.2% male vs. 63.8% female). In a similar pattern, clinical manifestation of CD and gender and age related features showed that the prevalence of celiac disease among females is four times more than males [14]. A female predominance might have some clinical implications; however, if a patient is suspected of CD, whether male or female, laboratory testing is recommended [7]. However, the pathophysiology underlying the higher rate of CD amongst female remains unclear, but the present findings support the hypotheses that gender biology plays a role in the disease development. Genetic factors, such as permissive HLA, gene variants on the X chromosome, and a higher prevalence in female relatives of the CD patients support our hypothesis [15–17].

Failure to thrive (FTT) is a typical manifestation of Celiac disease in pediatrics; however, a recent study in Wisconsin Hospital showed that 5% of children affected by celiac disease had BMI more than 95th percentile [18]. In the present study, 5.4% of the patients had BMI more than 95th percentile. Recently, being overweight and obesity has become more common amongst children with CD [19]. One possible mechanism might be the unpalatability of gluten-free foods which causes a preference for high caloric fat and protein foods [15]. Also, the increased prevalence of overweightness in children with CD can be explained by the global trend [20]. Semeraro et al. proposed an interesting hypothesis about the development of over-nutrition status in CD children which was the compensatory high energetic yield secondary to slow functional adaptation of the atrophic mucosa in these patients [21]. It was revealed that some children with celiac disease suffered from obesity and should be considered for long term treatments and follow ups.

A study in India showed that diarrhea, FTT, and distension are the most common symptoms in patients with CD [22]. Amongst Iranian adult patients with celiac disease, diarrhea, bloating, and weight loss were reported as the most common symptoms [13, 23]. However, in children in southeastern Iran, abdominal pain was the most common symptom with a prevalence of 41% [24]. In the present study, the most common symptom was abdominal pain followed by flatulence and constipation. We found that nonspecific symptoms like abdominal pain, flatulence and constipation might be the only presentations in children with CD, and should be taken into consideration. Recent guidelines recommend screening cases to find high risk population for CD, such as children with family history of CD like siblings or other first-degree relatives, children with other autoimmune diseases e.g. Type 1 diabetes, autoimmune hepatitis, and autoimmune thyroiditis, and those with chromosomal disorders associated with CD [25].

Reports from India showed that type 1 diabetes mellitus and hypothyroidism were the most common associated autoimmune conditions in children with CD [26]. One study in Australia showed that T1DM was the most common associated condition [27]. However, the present study showed that T1DM and hypothyroidism were the most common associated disease with CD amongst Iranian children. Honar et al. revealed that the prevalence of celiac disease in diabetes mellitus type 1 children in Iran was more common than that of America and Europe, but similar to that of Turkey [8]. Samasca et al. reported that about 30% of all patients with CD have one or more autoimmune disease while autoimmune diseases were found amongst 3 to 9.4% of the general population, but this association has not been completely understood, yet. A higher prevalence of autoimmune diseases amongst the relatives of CD children suggests that the genetic background is the main factor [28].

One study in Spain found that abnormal liver enzymes was seen in 40% of patients with CD [29]. In contrast, just 3% of the children in our study had abnormal liver enzyme, which might be due to increased intestinal permeability and alterations in gut microbiota, chronic intestinal inflammation, and genetic predisposition [30]. In our study, the patients with obesity had normal liver enzymes; consequently, we found that the elevated liver enzyme was not due to obesity and it might have been related to the CD itself. One study in Iran showed that the prevalence of CD in patients with nonalcoholic fatty liver disease was higher than the general population [31]. Also, another study showed that 15–55% of patients with CD had mild to severe liver complications. Hence, physicians should be aware that celiac disease could present itself with isolated liver enzyme elevation and evaluate liver enzyme in all patients with celiac disease [32]. However, it was shown that severity of clinical presentation, and the time of detection was associated with severity of histological damage [33]. Nowadays, physician’s knowledge on the heterogeneous presentation has increased, and also, available noninvasive screening tests of at-risk children make the diagnosis possible in earlier stages. Hence, we detected a lower incidence of elevated liver transaminase.

In this study, we evaluated the association of Marsh staging and clinical manifestations of celiac disease. We found that Marsh just correlated with long term fatigue. Among anthropometric measurements, it was shown that children’s height with CD in Marsh 3c was shorter than the other groups. Weir et al. found no significant association between histological findings and the clinical data, including gender, age, symptoms, and associated disorders, which might be due to the fact that duodenal involvement by CD in children is frequently patchy, more so than what is seen in adult patients with CD [34].

Despite many strengths, we had few limitations. It would have been much better if this study was conducted as a prospective trial, because we could have followed the effect of gluten free diet on clinical manifestations, and histologic findings of children with celiac disease in Iran. Also, it would be better if we had evaluated the patients’ compliance in using GFD.

## Conclusion

The present study showed that the most common GI symptoms in the children with CD, are abdominal pain, flatulence and constipation, and the most common extra intestinal manifestation are bone pain, long term fatigue and anemia. The most common associated comorbidities with CD are type 1 diabetes mellitus and hypothyroidism. From all the manifestations, flatulence, chronic diarrhea, paresthesia in hands and feet were observed more amongst male patients. Conducting a prospective study is recommended to evaluate the effect of gluten free diet on clinical manifestations, and histologic findings of children with celiac disease in Iran.

## Data Availability

The datasets used and/or analyzed during the current study are available from the corresponding author upon request.

## Abbreviations

CD: Celiac disease
GI: Gastrointestinal
ACG: American College of Gastroenterology
TTG: Anti-tissue transglutaminase
SUMS: Shiraz University of Medical Sciences
BMI: Body mass index
T1DM: Type 1 diabetes
